# The Sheltering of Unwanted Cattle, Experiences in India and Implications for Cattle Industries Elsewhere

**DOI:** 10.3390/ani8050064

**Published:** 2018-04-26

**Authors:** Uttara Kennedy, Arvind Sharma, Clive J. C. Phillips

**Affiliations:** 1 RSPCA Queensland, Wacol, QLD 4076, Australia; uttaram@yahoo.com; 2 Centre for Animal Welfare and Ethics, School of Veterinary Science, University of Queensland, Gatton, QLD 4343, Australia; arvind.sharma@uqconnect.edu.au (A.S.); c.phillips@uq.edu.au (C.J.C.P.)

**Keywords:** cow, shelter, gaushala, welfare, animal, India, dairy, slaughter, cattle

## Abstract

**Simple Summary:**

The cow has evolved to become one of the most important symbols of Hindu identity, often synonymous with religious or nationalistic sentiment and pride. The issue of protecting and revering the cow has been the cause of much unrest, violence and vigilantism; this continues to be the case even in the present day. Nowadays the law bans cow-slaughter in a majority of Indian States. A direct result of these anti-slaughter laws is a large population of abandoned, aged or otherwise unproductive cattle housed in cattle-shelters that often face inadequacy of space, infrastructure, skilled labour and lack of financial and veterinarian support. Many members of the Indian community do not necessarily look upon this phenomenon negatively, since they view historical events spanning three millennia of Indian history from the perspective of cow-symbolism. We discuss the moral, social and welfare ramifications of this unique phenomenon of nationwide animal worship and protection, exploring whether such strategies could find application in the Western milk, meat and egg production context.

**Abstract:**

Reverence for the cow has been a centerpiece of Hindu culture, the roots of which can be traced back to the Indus Valley Civilization around 3000 BCE. Historical and anthropological literature demonstrates how over the millennia the animal’s status as a religious symbol steadily increased and the concept of its sanctity grew in complexity, becoming deeply entrenched and assuming a core identity of the religion. The cow has also been used as a symbol of political opposition to external influences and invading powers. Nowhere else in the world has an animal maintained such divine significance into modern day. This literature review explores the interplay of complex cultural, religious, social and political factors that led to the phenomenon of the sacred cow, a ban on its slaughter and the advent of the modern gaushala. The review also discusses the moral implications of preservation of animal life past their commercial use, the impact on their welfare and need for objectively assessing whether there is a place for such strategies in other animal industries worldwide.

## 1. Introduction

Beginning about 4000 years BCE, warriors from the central steppe region of Asia invaded India, bringing with them their habits of expropriating cattle from villages that they plundered. Cattle became in scarce supply as food producers [1], and through a complex interplay of cultural and socio-economic factors, reverence for the cow became a centerpiece of Hindu culture; the roots of this reverence can be traced back to the Indus Valley Civilization around 3000 BCE [2,3,4,5]. Over the millennia the cow’s status as a religious symbol steadily increased and the concept of its sanctity grew in complexity, becoming deeply entrenched and assuming a core identity of the region. By 1500 BCE a ritualistic and sacrificial role for cattle was evident in the Vedic literature.

In later centuries, the divine symbol of the cow came to be used as a symbol of Indian national identity for rallying against external invading powers [2,4,6,7]. Nowadays, almost 30% of the world’s cattle population of approximately 1.4 billion [8] resides in India. Because of their sanctity in the Hindu religion, they are not usually slaughtered for meat, but are used for production of milk, milk products and faeces. The Indian political, religious and cultural landscape is still dominated by cow worship in many forms. Cow-dung, urine, milk, milk products [9,10] and even dust from cow-prints [3,6] are venerated. Mainstream Indians maintain a strong repulsion to beef and widely celebrate cow-centric festivals such as Govardhan Puja and Gopashtami [3,6,9]. Nowhere else in the world has an animal maintained such consistence and deep-rooted divine significance into modern day [9].

This review explores the interplay of cultural, religious, social and political factors that led to the phenomenon of the sacred cow, the ban on its slaughter and the advent of the modern gaushala. It further discusses the morality, welfare impact and socio-economic importance of gaushalas in India as well as animal protection in the wider context of animal industries throughout the world.

## 2. A Historical and Chronological Perspective

About two million years ago the first members of a new genus of grazing animal, Bos, began to appear in northern India and established themselves as wild cattle or aurochs in India and central East Asia. About half a million years ago in the Pleistocene period a distinct subtype of *Bos* cattle developed in the Indian subcontinent after the Ice ages—the humped *Bos primigenius namadicus*. This was the forebear of the modern zebu cattle, which came to predominate in the Indian subcontinent and became known as *Bos indicus* [1].

The oldest evidence of cattle assuming any kind of symbolic role can be traced back to the temples and friezes of the Mesopotamian Civilization, which is thought to have influenced the Harappan Civilization of the Indus Valley [2,3,5,11]. Centuries later, as the agricultural Harappan Civilization declined and fragmented, the pastoral Aryans descended from the northwest. By 1500 BCE the Aryans had composed and written the Vedas, a large body of literature that is considered the original sacred scripture of modern day Hinduism [2,3]. Vedic writings demonstrate not only pastoral and economic importance of cattle but in equal measure the ritualistic and sacrificial role of the animal [3,5,6,9]. One of the texts for example, cites the cow as the ‘all-producing, all-containing universe’ [9] and its sacrifice was considered to be essential to the very order of cosmic creation of the universe [4]. By the end of the Vedic period (800–600 BCE), the metaphoric, literary and figurative cow of the Indus Valley and early Vedic times had transformed into a literal object of sanctity, to be protected and revered in its own right [2,3,4]. Subsequent treatises, like the Upanishads, elaborated on the old Vedic teachings. Simultaneously, emerging sects like Jainism and Buddhism added to the general concept of inviolability of animals (including the cow), by introducing the philosophy of ahimsa (non-violence), karma and the possibility of human re-incarnation as animals and vice versa [2,3,4].

Buddha’s message spread great distances during the rule of King Asoka in the 2nd century BCE, and for a time ahimsa or non-violence became the law of the land [2,3,9]. Even traditional Hinduism started incorporating and legitimizing the concepts of ahimsa and compassion by applying it specifically to the cow [3]. Animal-shelters (panjarapoles) and cow-homes (gaushalas) emerged all over the country to provide official religious sanction to the protection of cattle and increasing restrictions on their slaughter. The Jains to this day have ahimsa at the very core of their philosophy: espousing practices like vegetarianism, avoidance of consumption of root vegetables and shunning agricultural practices that necessitate destruction of pests [3].

The Mughal invasions of India started in the 11th Century. The cow, already a centerpiece of Hindu philosophy, became a rallying point between the invading beef-eating culture and native communities [2,3,9,11,12]. Muslim kings like Babar and Akbar in the 14th Century passed edicts forbidding cow slaughter in an effort to promote good will and reconciliation with native populations [2,9]. Other leaders like Aurangzeb slaughtered cows as a deliberate symbol of disregard and subjugation of Hindu populations [9]. The Maratha King Shivaji (1627–1680) was the first campaigner of cow protection by using the cow as a political symbol to unite the Hindus against the Muslim invaders [9].

Nationalistic sentiment continued to grow during the two hundred years of the British Raj [2,12]. The first large-scale revolt against the Raj in 1857 was in part due to a rumor that the British used tallow from beef to grease cartridges used by Hindu soldiers in their rifles [10,13]. Then, in 1881 the first nation-wide cow-protection movement was started by a renowned religious leader of the time, Swami Dayanand Saraswati [4,14]. Many other Hindu-centric movements followed, one of which was the Rashtriya Swayam Sevak Sangh, which started in 1925 and still retains right-winged cow-centric political discourse [6]. The foremost leader of the Indian struggle for independence, Mahatma Gandhi founded his own cow-protection agencies based on compassion and welfare [4]. As a reaction to these emotionally charged cow-protection movements, the British government passed the first Indian animal cruelty legislation to shift the focus from a nationalistic and religious emphasis to a more scientific and economic perspective of the cow. Commissions were appointed and cattle husbandry was promoted on scientific lines through the establishment of research institutes and dissemination of knowledge about model cattle farms [4]. 

Several accounts of travellers to India during the 14th-17th centuries mentioned the presence of animal homes in the form of “gaushalas” which sheltered just cattle and “panjarapoles” which sheltered all types of animals, akin to modern day animal shelters. The gaushalas had a mainly Hindu association and the panjarapole a Jain association. Their exponential growth during the time of the British Raj probably derived from growing nationalistic sentiment antagonistic to British occupation [2].

The most popular of all Hindu Gods, Krishna, has been widely loved, cherished, painted and sung of in his role as a protector of cows—a cow herder living amongst pastoral villagers [2,3]. In recent centuries, the largest cult dedicated to Krishna-devotion was founded and promoted by the philosopher Vallabhacharya in the western regions of Gujarat and Rajasthan—an area that even today contains the largest number of vegetarians, cow-worshippers and gaushalas in India.

It was against a backdrop of strong nationalistic feelings mingled with religiously charged sentiments that India gained independence as a secular country in 1947 [5,6,15]. A Constituent Assembly was established to draft India’s first Constitution. From records of its deliberations [5,7], it is evident that the issue of legally banning cow slaughter was fraught with religious and emotional sensitivities. Staunch Hindus argued that cow slaughter went against the very core of their religion, while Muslims argued that any ban on cow slaughter infringed on their right to earn a livelihood as butchers, leather-makers, tanners and slaughter-men. A third secular faction argued that a secular Constitution should not contain prejudices or sanctions for specific religious beliefs [5]. So charged was the atmosphere that riots broke out intermittently all over the country during these deliberations [6]. The final Constitution contains an Article inserted into the Directive Principles of State Policy, not legally enforceable but recommending that the States frame their own enforceable laws to protect cows from slaughter [7,16,17,18]:

“The State shall endeavor to organise agriculture and animal husbandry on modern and scientific lines and shall, in particular, take steps for preserving and improving the breeds, and prohibiting the slaughter, of cows and calves and other milch and draught cattle.”

Gradually State laws came into effect all over the country and several prosecutions followed, mainly against cattle buyers and sellers, hide merchants, butchers and other such stakeholders in the cattle industry [5,6,17]. Post-independence India continued to see periodic riots, agitations and even killings in the name of the sacred cow. In 1966, several conservative Hindu political parties such as the aforementioned Rashtriya Swayam Sevak Sangh, sections of the Jain community, the Arya Samaj community, the Bhartiya Jana Sangh and others joined forces to march and form a Committee for Cow-Protection. It was perhaps the largest demonstration in history to demand a complete nation-wide ban on cow-slaughter. Several people were killed, properties were damaged and agitators were jailed as a result of the march [6]. Modern day sees ample media reports of vigilantism by self-proclaimed cow protectors, biased court prosecutions and blocking of cattle slaughter and transport licenses [5,7,18]. In 2005, self-proclaimed cow protection agencies lynched members of the dalits community, who traditionally hold the job of skinning cattle carcasses [17]. The year 2016 witnessed alleged cattle traders and their families being stripped naked and beaten in public [19,20]. Right-winged Hindutva actions are increasingly backed by constitutional Supreme Court support [7,17].

**Figure 1:**
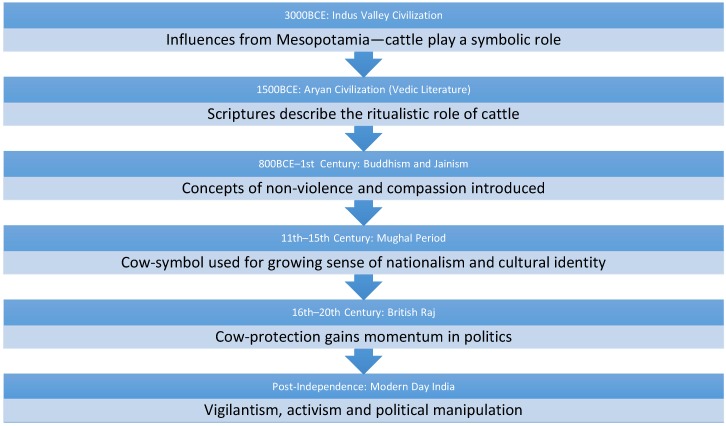
Chronology of Indian historical events surrounding cow-symbolism.

In summary, there have been 3000 years of events contributing to cow-centrism, cow-worship and a complete countrywide legal ban on its slaughter (Figure 1).

## 3. The Modern Indian Gaushala and India’s Surplus Cattle

For the Hindu community, the gaushala is inextricably tied to religion: many are directly associated with temples or religious institutions. Even those that are not, actively encourage rites, rituals and celebration of cow-centric festivals. Either out of veneration or fear of a backlash from activists, rather than slaughter or sell cattle that are past their productive life or suffering from disease or debilitation, villagers often abandon these animals to the streets. These cattle then remain as stray or get picked up by neighbouring gaushalas rather than being slaughtered [6,21,22]. Gaushalas also often see an increased influx of cattle and small ruminants rescued from ritual slaughter during Muslim festivals [2].

The Report of the National Commission on Cattle [23] determined that there were three thousand gaushalas in India, maintaining over 600,000 cattle, about one seventh of the total Indian cattle population. However, this may be an underestimate, Khanna [24] puts the countrywide estimate at around four thousand gaushalas. There is a dearth of comprehensive studies on urban animal health in India [21] and a true estimate for country-wide numbers of stray cattle or gaushalas is lacking.

In the 1960s, when India experienced severe drought and famine, its agricultural economy and the issue of useless cattle competing with humans for scarce resources sparked the interest of several ecologists and anthropologists from around the world [3]. Harris (originally published in 1966) [25] propounded that the inviolability of the cow had a positive economic and social benefit. He explained that the absence of commercial beef enterprises made beef a more accessible source of protein to minority and marginal communities that would otherwise be cut off from expensive commercially available meats. Recent journal articles and news reports [17,18] support this argument. However, Simoons et al. [15] countered that these surplus cattle compete with humans for land and resources, cause substantial damage to crops, impede breed improvement [22], cause soil erosion from overgrazing and are a wasted resource of beef and leather. The issue generated considerable debate from economic and husbandry specialists on both sides, and came to be known as the “sacred-cow controversy” in the literature [3].

## 4. Welfare Issues for India’s Surplus Cattle

With one of the highest cattle populations per capita in the world, maintaining inefficient cattle production systems has been particularly difficult in the face of increased human population. Many abandoned cattle can only survive by scavenging food and less than 20% of their feed is suitable for consumption [1]. The absence of refuse collection in most of India allows cattle to recycle waste foods, but they also consume large quantities of indigestible and potentially toxic materials in their search for food residues. Many forage for food in exposed garbage dumps, consuming large amounts of plastic bags that contain discarded human food. The complex cow stomach cannot expel these bags, which remain trapped inside, rendering the animal unable to eat and slowly starving to death. Post mortems have revealed up to 40 kg of plastic in the rumen or fore-stomach of stray cattle, where the plastic is trapped [25]. There it forms an indigestible mass, effectively reducing the volume available for the cow’s own digestive system.

Many cattle are smuggled across India’s borders for slaughter or travel long distances to those states permitting slaughter (in particular Kerala). They are often sold clandestinely so as not to attract the attention of both lay observers as well as law enforcers [6,15] and as a result their transport, lairage and slaughter are driven underground. The conditions that these animals are kept, transported and handled remains completely unregulated. It is estimated that compared to 3,600 legal slaughter houses (for buffaloes, small ruminants and other commercial animals excluding cattle), there are 32,000 unlicensed ones [17] that are quite possibly illegally slaughtering cattle for export or leather. It is not possible to get official data on this type of clandestine activity, and studies of Bangladeshi abattoirs and livestock markets have found that many of the cattle were legally or illegally exported from India by trek, truck or train [26,27]. One estimate puts the numbers at 1.7 million cattle get exported annually, the majority of which are slaughtered for meat production [28]. Many of these animals show severe nose and tail injuries [27], dehydration and metabolic exhaustion [26] hyperthermia and death during transport [28].

Gaushalas often face with an inadequacy of skilled labour, financial constraints and lack of veterinarian support [11,22]. The herds, already a cull by-product of a commercial population often suffer from malnutrition, further compounding pre-existing reproductive disorders like anoestrous, repeat breeding, uterine infection, cervicitis, pre and postpartum vaginal prolapse, retention of placenta, dystocia and mastitis [22]. Brucellosis, a zoonotic disease, is endemic in Indian cattle populations; conditions of intensive housing and frequent movement of cattle are particularly conducive to the spread of this disease [29]. A reluctance to cull infected animals means that it is virtually impossible to control or eradicate this disease in Indian herds. Those that are culled usually enter the gaushalas, but often are not screened for this disease and become a health hazard to both the workers and other cows. Many cattle housing facilities, including commercial dairies have insufficient space, little to no pasture land, poor ventilation and hygiene [11,21].

Although individual gaushalas have been studied for incidences of disease outbreaks [30,31], multidimensional animal welfare assessments on multiple gaushalas have never been carried out. The literature over the last 50 years regularly documents the social, economic, religious and political aspects of cattle utilization and conservation: numerous studies [22,24], including the Report of the National Commission on Cattle [23], have advocated using gaushalas as centers for breed improvement, increased productivity and breed conservation. Yet, none of the studies have considered cattle welfare; furthermore the quality of life of the cattle themselves has not been holistically investigated.

## 5. The Morality of Sheltering Cattle in India

The sentiment that many stakeholders have towards cow welfare in India is similar to that in many western countries towards dogs. In the west, “no-kill” policies are often applied to dog shelters. A UK-based charity, Dogs Trust states on their website [32]:

“A non-destruction policy is central to our ethos. We love dogs and we’re dedicated to saving those whose lives are at risk. Once a dog comes into our care, we treat him or her as we would a much-loved family pet. We’re a dog charity—we’re here to save dogs, not to kill them.”

The appeal of such policies may lie in the fact that they take a purely moral stand, without giving in to economic or logistical arguments. The gaushalas of India are founded on similar moral arguments and are largely looked upon with respect and admiration as pillars of moral, spiritual and religious principles. In the West, central to the companion animal sheltering philosophy is the issue of ownership [33]. People are clearly identified as owners with legal responsibilities, that require them to compensate anyone injured or whose property does the companion animal damage. Shelters encourage those relinquishing their companion animals to identify as the former owner, and transition of that ownership to the shelter must be accompanied by payments for future costs. A similar situation exists for cattle in India (author’s observations). A fee of about US $75 for future costs accompanies cattle that are relinquished to gaushalas. As in the West, many cattle are rounded up by Non-governmental organization (NGO)’s if they are found on the streets and taken to shelters (particularly in Himachal Pradesh, Punjab and Haryana). In other states, notably Gujarat, Rajasthan and Maharashtra, street cattle are not relinquished directly to gaushalas; they enter a municipal pound and are then taken to the gaushalas if owners do not claim them. Similarly, horses that have surpassed their useful life are not euthanized but kept in shelters, albeit in much smaller numbers than cattle. Stray dogs are usually subject to trap, neuter and release, and only a few are sheltered for adoption. Thus the same system of sheltering exists in India for many animal species as for dogs in Western countries, except that in India, animals are rarely if ever legally euthanized. The implications of animal sheltering being extended to animals other than dogs in India and the rarity of euthanasia is very great on the country’s infrastructure. Even if welfare isn’t always of the highest standard, the community can be comfortable in the knowledge that animals that have advanced past their use to humans are allowed to live out their natural lifespan in a safe environment. Such considerations are increasingly prominent in Western society, where the premature culling of male dairy calves raises serious concern [34]. The Indian experience documented in this paper may inform future debate in the West about how to deal with unwanted cattle, including male calves from the dairy herd. However, Western philosophy places more emphasis on animals’ welfare and less on animals’ rights to life [35]. This was driven by religious beliefs, since the Abrahamic faiths belief that animals were put on earth for man’s benefit [36]. As these beliefs wane there may be more scope to consider that the sheltering of cattle after their useful life has a role in any society that cares about its animals. Not only do people in the West care about the animals in their society, they are sufficiently affluent to be able to shelter animals without any use effectively, avoiding the welfare problems that exist in Indian gaushalas. However, whilst an economic imperative of making as much money from animals prevails in the West, with the involvement of major industries, it will be difficult for people to make their voice heard in wanting a longer life for production animals.

## 6. Conclusions

Through events spanning over three thousand years, the cow has evolved to become perhaps the most important symbol of Hindu identity, often synonymous with religious or nationalistic sentiment and pride. The issue of protecting and revering the cow has been the cause of much unrest, violence and vigilantism; this continues to be the case even in the present day. On a broader perspective, it is a unique case of prevention of animal wastage in the contemporary world where factory farming has spread all over the Western world, commodifying animals rather than treating them as sentient beings. In present times, most States of India have legally banned the slaughter of cows, resulting in a largely unproductive surplus cattle population either roaming the streets or living in animal shelters. Many members of the Indian community do not necessarily look upon this as a negative, since they place intrinsic value in saving the animals from premature slaughter. Gaushalas or cow-homes have been present for several centuries, serving different functions at various times in history. The modern-day gaushala is often seen to be ill equipped in terms of manpower, resources, technology and expertise. Yet, the effect of abandonment, clandestine slaughter or confinement in gaushalas has not been studied from the specific point of view of animal welfare. As they contain a significant proportion of the nation’s cattle, the sheltering of cattle in gaushalas deserves full and comprehensive attention, not just for its national importance, but also for its international importance when many are questioning the wastage of cattle, in particular male calves, in western society.
